# Assessing reproductive status in elasmobranch fishes using steroid hormones extracted from skeletal muscle tissue

**DOI:** 10.1093/conphys/cot028

**Published:** 2013-11-04

**Authors:** Bianca K. Prohaska, Paul C. W. Tsang, William B. Driggers, Eric R. Hoffmayer, Carolyn R. Wheeler, A. Christine Brown, James A. Sulikowski

**Affiliations:** 1 Department of Marine Sciences, University of New England, 11 Hills Beach Road, Biddeford, ME 04005, USA; 2 Department of Molecular, Cellular and Biomedical Sciences, University of New Hampshire, 129 Main Street, Durham, NH 03824, USA; 3 National Oceanic and Atmospheric Administration, National Marine Fisheries Service, Mississippi Laboratories, 3209 Frederic Street, Pascagoula, MS 39567, USA

**Keywords:** Steroid hormones, reproductive status, elasmobranch, non-lethal sampling

## Abstract

Elasmobranch fishes (sharks, skates, and rays) are particularly susceptible to anthropogenic threats, making a thorough understanding of their life history characteristics essential for proper management. Historically, elasmobranch reproductive data have been collected by lethal sampling, an approach that is problematic for threatened and endangered species. However, recent studies have demonstrated that non-lethal approaches can be as effective as lethal ones for assessment of the reproductive status of an animal. For example, plasma has been used to examine concentrations of steroid hormones. Additionally, skeletal muscle tissue, which can be obtained non-lethally and with minimal stress, can also be used to quantify concentrations of steroid hormones. Skeletal muscle progesterone, testosterone, and estradiol concentrations were determined to be statistically significant indicators of reproductive status in the oviparous *Leucoraja erinacea*, the yolk-dependent viviparous *Squalus acanthias*, and the yolk-sac placental viviparous *Rhizoprionodon terraenovae*. The results of the present study demonstrate that steroid hormones present in non-lethally harvested skeletal muscle tissue can be used as reliable indicators of reproductive status in elasmobranchs.

## Abstract (precis)

Understanding the life history of elasmobranch fishes (sharks, skates, and rays) is essential for their management. This study investigated the utilization of steroid hormone concentrations in non-lethally harvested elasmobranch skeletal muscle to assess reproductive status.

## Introduction

The life history characteristics of many elasmobranchs, such as slow growth and late age at maturity, make these fishes particularly susceptible to overexploitation (Dulvy *et al.*, 2003). As a result, anthropogenic threats, such as direct and indirect commercial fishing, have led to significant population declines in numerous elasmobranch species (Dulvy *et al.*, 2008). To manage elasmobranchs effectively, a comprehensive understanding of their life history characteristics is needed. However, data on these characteristics are lacking for many species (Castro *et al.*, 1999; Walker, 2004; Pinhal *et al.*, 2008; IUCN, 2011). For instance, essential life history characteristics needed for proper management include age/size at maturity, gestation length, and reproductive cyclicity (Walker, 2004, 2005). When this information is incorporated into fisheries models and species assessments, insight can be gained into when, where, and how often populations are reproducing, which can ultimately aid in reducing their decline through the development of management protocols that augment fishery practices (Walker, 2004, 2005). For example, the management of blacknose sharks within the USA was directly affected and improved by the inclusion of data detailing regionally distinct reproductive periodicity (NMFS, 2011).

Lethal sampling has historically been considered the most effective approach for collecting life history information from elasmobranchs, particularly because it is quick and provides a comprehensive set of data (Heupel and Simpfendorfer, 2010). More recently, in response to species declines and ethical concerns, elasmobranch research has moved towards conservation-minded sampling approaches (Sulikowski *et al.*, 2007; Heupel and Simpfendorfer, 2010; Hammerschlag and Sulikowski, 2011).

Among the non-lethal approaches to studying the reproductive biology of elasmobranchs, such as intrauterine endoscopy (Carrier *et al.*, 2003) and ultrasonography (Daly *et al.*, 2007), analysis of plasma hormones is the most widely used (e.g. Kneebone *et al.*, 2007; Sulikowski *et al.*, 2007; Henningsen *et al.*, 2008). In particular, plasma levels of steroid hormones are correlated directly with reproductive events in elasmobranchs, such as the onset of maturity (Gelsleichter *et al.*, 2002; Sulikowski *et al.*, 2006) and reproductive activity (Sulikowski *et al.*, 2004; Kneebone *et al.*, 2007). Although plasma steroid hormone analysis has been conducted on many species of elasmobranchs spanning multiple reproductive modes (e.g. Gelsleichter *et al.*, 2002; Sulikowski *et al.*, 2006; Awruch *et al.*, 2008; Henningsen *et al.*, 2008), obtaining blood can be stressful for the animal because of the significant amount of handling time and prolonged removal from the water (Skomal, 2007). Following the success of extracting steroid hormones from bodily materials other than plasma and correlating concentrations to reproductive events in non-elasmobranch vertebrates (Heppell and Sullivan, 2000; Mansour *et al.*, 2002; Kellar *et al.*, 2006; Barnett *et al.*, 2009), a new non-lethal approach has recently been developed that is potentially less stressful, because it does not require the animal be restrained or removed from the water, and it can be conducted rapidly. Prohaska *et al.* (2013) investigated the efficacy of extracting steroid hormones from the skeletal muscle tissue of elasmobranchs using two reproductive modes, the yolk-sac placental viviparous *Rhizoprionodon terraenovae* and the yolk-dependent viviparous *Squalus acanthias.* Steroid hormones were successfully extracted and quantified from skeletal muscle tissue, but more importantly, fluctuations in these hormones are correlated with gestational stage (Prohaska *et al.*, 2013).

It is critical that effective non-lethal protocols for studying elasmobranch reproductive biology are thoroughly tested and validated prior to the shift towards their strict use in research, similar to what occurred for marine mammals in the USA (NMFS, 1972; Heupel and Simpfendorfer, 2010; Hammerschlag and Sulikowski, 2011). As a result of the diversity of reproductive modes in elasmobranchs, oviparity (egg laying), yolk-dependent viviparity (live birth of yolk-dependent embryos), and yolk-sac placental viviparity (live birth of embryos with an initial yolk-sac followed by a placental attachment; Wourms, 1977), coupled with the promising results of Prohaska *et al.* (2013), the objectives of the present study were as follows: (i) to attain a more comprehensive understanding of muscle steroid hormones in *R. terraenovae* and *S. acanthias*; (ii) to determine whether this approach is appropriate for studying the reproductive biology of oviparous elasmobranchs using *Leucoraja erinacea*; and (iii) to determine whether skeletal muscle steroid hormones can be used as non-lethal indicators of reproductive status in elasmobranchs.

## Materials and methods

### Specimen collection

Female *R. terraenovae* and *S. acanthias* were captured using the same methods and in the same locations as previously described by Prohaska *et al.* (2013). Briefly, *R. terraenovae* were captured by bottom longline in the northern Gulf of Mexico in an area centred around 88.812°W and 27.887°N, while *S. acanthias* were captured by bottom trawl and gill net in the US Northwest Atlantic in an area centred around 70.115°W and 42.471°N. The aforementioned fishing methods were conducted for a maximum of 1 h, with blood sampling taking place immediately after capture, to reduce the potential for stress hormones to interfere with sex steroid hormone concentrations. Female *L. erinacea* were captured by bottom trawl in November 2012 in an area centred around 70.466°W and 42.615°N. After capture, *L. erinacea* were maintained in an insulated livewell containing ambient surface seawater. To ensure that water quality was maintained, frequent water changes occurred during the ∼1.5 h return to the dock. All live-captured *L. erinacea* were transported from the dock in an aerated 833 l insulated livewell to the University of New England's Marine Science Center (∼2 h) and housed in a 3785 l, 2.4 m diameter hexagonal tank with an open flow-through seawater system with a turnover rate of 38 l min^−1^. Animal husbandry of *L. erinacea* followed the protocols of Palm *et al.* (2011). Additionally, skates were palpated daily for up to 3 weeks to assess presence or absence of egg cases. Immediately prior to obtaining internal morphological data, *L. erinacea* were killed by lethal pithing.

### Sampling

Sampling of *R. terraenovae*, *S. acanthias*, and *L. erinacea* followed the same protocols as those described by Prohaska *et al.* (2013). Briefly, at the time of sampling, an 8 ml aliquot of blood was collected and stored at 4°C for up to 24 h. Blood was then analysed for haematocrit prior to being centrifuged at 1242 g for 5 min. Plasma was then removed and stored at −20°C until steroid hormone analysis. The following morphological parameters were recorded: mass (in kilograms), fork length (FL; in sharks), disc width (DW; in skates) and natural total length (TL), all of which were measured to the nearest centimetre over a straight line along the axis of the body. Additionally, ovary and oviducal gland mass (in grams), oviducal gland width (in millimetres), and follicle diameter (in millimetres) were recorded. From sharks, embryo sex and stretch total length (STL; in millimetres), measured to the nearest millimetre over a straight line along the axis of the body, were also recorded. A 5 g white skeletal muscle tissue sample was then collected from behind the second dorsal fin from all sharks, and from the mid-point of the right pectoral fin from all skates, and immediately stored at −20°C until analysis (Prohaska *et al.*, 2013).

### Plasma steroid hormone extraction

Progesterone (P_4_), testosterone (T), and 17β-estradiol (E_2_) were extracted from all plasma samples following the methods of Tsang and Callard (1987) and Sulikowski *et al.* (2004). Briefly, each plasma sample was extracted twice with 10 volumes (5 ml) of ethyl ether (ACS grade), and the organic phase was evaporated at 37°C under a stream of nitrogen. Extracts were reconstituted in phosphate-buffered saline (PBS) containing 0.1% gelatin. Prior to extraction, each sample was spiked with 1000 counts min^−1^ of tritiated P_4_, T, or E_2_ (Perkin Elmer, Waltham, MA, USA) to account for procedural loss.

### Skeletal muscle tissue steroid hormone extraction

The hormones P_4_, T, and E_2_ were extracted from all white skeletal muscle tissue samples following the protocol of Prohaska *et al.* (2013). Briefly, 2 g of white skeletal muscle tissue from each individual were homogenized with 8 ml of cold PBS and divided into 500 μl quadruplicate aliquots. All replicates were incubated at 50°C for 15 min prior to extraction with 10 volumes (5 ml) of 2:1 chloroform/methanol (ACS grade; histology grade). The organic phase was evaporated at 37°C under a stream of nitrogen before reconstitution in 1 ml of 70% methanol (histology grade) and kept at −20°C for 24 h. Samples were then centrifuged at 962*g* for 10 min at 4°C, before decanting and evaporating the methanol phase at 37°C under a stream of nitrogen. Dried extracts were reconstituted in PBS containing 0.1% gelatin. To account for procedural loss, two of the four replicates for each muscle sample were cold-spiked with the corresponding non-radiolabelled steroid hormone (Steraloids, Inc., Newport, RI, USA) prior to extraction.

### Radioimmunoassay

Plasma and muscle steroid hormone concentrations were determined by radioimmunoassay, following a modified protocol from Tsang and Callard (1987). Non-radiolabelled P_4_, T, and E_2_ (Steraloids, Inc.) were used to make stock concentrations of 80 μg ml^−1^ for P_4_ and T, and 6.4 μg ml^−1^ for E_2_ in absolute ethanol (ACS grade). The P_4_, T, and E_2_ antibodies (Gordon D. Niswender, Colorado State University, Fort Collins, CO, USA) were diluted to final concentrations of 1:2500, 1:10 000, and 1:18 000, respectively. Tritiated hormones and antibodies were added to the reconstituted plasma and muscle samples using PBS containing 0.1% gelatin to bring the total assay volume to 400 μl. After incubation at 4°C for 24 h, free hormone was separated from bound hormone by the addition of a carbon (0.2%; Acros Organics, Fairlawn, NJ, USA) and dextran 70 (0.02%; Tokyo Chemical Industry Co., Ltd, Tokyo, Japan) suspension, and centrifuged at 1242*g* for 10 min at 4°C. The supernatant was combined with 3.5 ml of Ecolume (MPO Biomedicals, Solon, OH, USA), and the radioactivity was detected by a Perkin Elmer Tri-Carb 2900TR liquid scintillation analyzer (Waltham, MA, USA). Final concentrations were corrected for procedural loss using individual sample recoveries. When calculating the mean and standard error (±SEM) of plasma and muscle steroid hormone concentrations per stage, any value that was non-detectable was assigned the lowest possible concentration detectable by the assay for the aliquot utilized.

### Statistical analysis

Data obtained from 10 *R. terraenovae* by Prohaska *et al.* (2013) were pooled with all *R. terraenovae* data collected in the present study for analysis. Additionally, statistical analyses were conducted on data obtained from 31 *S. acanthias* by Prohaska *et al.* (2013). Linear regressions were performed on plasma and skeletal muscle P_4_, T, and E_2_ concentrations by species, *R. terraenovae* and *L. erinacea*. One-way ANOVAs were performed for *R. terraenovae* and *S. acanthias* on plasma and skeletal muscle P_4_, T, and E_2_ concentrations by gestational stage, followed by Tukey's *post hoc* test. If variables failed tests of normality or homogeneity of variance, the data were Box–Cox transformed. If transformed variables still violated the assumptions, the non-parametric Kendall's τ rank correlation or a Kruskal–Wallis rank sum test was performed instead of linear regression or one-way ANOVA, respectively. Multiple regression analyses were conducted by species to generate mathematical equations that may be used to predict each of the following morphological parameters: maximal follicle diameter (MFD), ovary mass (OM), and oviducal gland mass (OGM) for both sharks and skates, and embryo STL for sharks, using skeletal muscle P_4_, T, and E_2_ concentrations as explanatory variables. Prior to regression analyses, all skeletal muscle P_4_, T, and E_2_ concentration data were log transformed to meet the assumptions of normality and homogeneity of variance. Multiple regression equations were generated by backwards selection of a fully interactive polynomial model, including all interactions between the first-order hormone terms and all first-order hormone terms squared, cubed, and to the fourth power. Any term that was found to be non-significant (*P* > 0.05) was removed from the model until all highest-order terms were significant. If co-linearity was detected in a model, all first-order terms within that model were centred, and the multiple regression equation was re-generated following the same backwards selection procedure. All data were analysed using R 2.15.2 (R-Core Development, 2012). All tests were considered significant at α le; 0.05.

## Results

### 

#### Rhizoprionodon terraenovae

A total of 24 female *R. terraenovae* (FL, 69–92 cm; 1.6–7.9 kg) were sampled and assigned to the following discrete reproductive stages: five immature, four pre-ovulatory, six mid-gestation (containing embryos of STL 100–142 mm), and nine in late gestation (containing embryos of STL 314–364 mm; see Table 1 for gestational stage details). In addition, data from 10 female *R. terraenovae* collected by Prohaska *et al.* (2013) were combined with the present data set, boosting the overall sample size (*n* = 34). These data included female *R. terraenovae* assigned to the following discrete reproductive modes: one pre-ovulatory, two early gestation (containing embryos of STL 28–55 mm), four early to mid-gestation (containing embryos of STL 56–83 mm), and three mid-gestation (containing embryos of STL 85–139 mm). The overall mean recoveries of plasma and muscle P_4_, T, and E_2_ were 74, 91, and 78% and 52, 47, 51%, respectively. The mean intra-assay coefficients of variation for *R. terraenovae* plasma and muscle P_4_, T, and E_2_ assays were 8, 7, and 5% and 10, 8, and 7%, respectively. The mean inter-assay coefficients of variation for *R. terraenovae* plasma and muscle P_4_, T, and E_2_ assays were 12, 10, and 12% and 12, 13, and 12%, respectively.

**Table 1.** *Rhizoprionodon terraenovae* morphological data

Stage	OM (g)	OGM (g)	MFD (mm)	Embryo STL (mm)	*n*
Pre-ovulatory	24 ± 4.5	2.7 ± 0.4	20 ± 1.3		5
Early gestation			4.8 ± 0.9	37.7 ± 9.8	2
Early to mid-gestation	5.8 ± 1.5	1.4 ± 0.3	6.9 ± 1.1	66.8 ± 4.2	4
Mid-gestation	3.6 ± 0.3	1.1 ± 0.2	4.3 ± 0.2	126 ± 6.8	9
Late gestation	20 ± 3.2	2.9 ± 0.2	19 ± 1.0	338 ± 5.7	9

Values are means (± SEM), by reproductive stage; *n* represents sample size. Abbreviations: embryo STL, stretch total length; MFD, maximal follicle diameter; OGM, oviducal gland mass; and OM, ovarian mass. The table includes data from the 10 *R. terraenovae* sampled by Prohaska *et al.* (2013).

**Figure 1.**
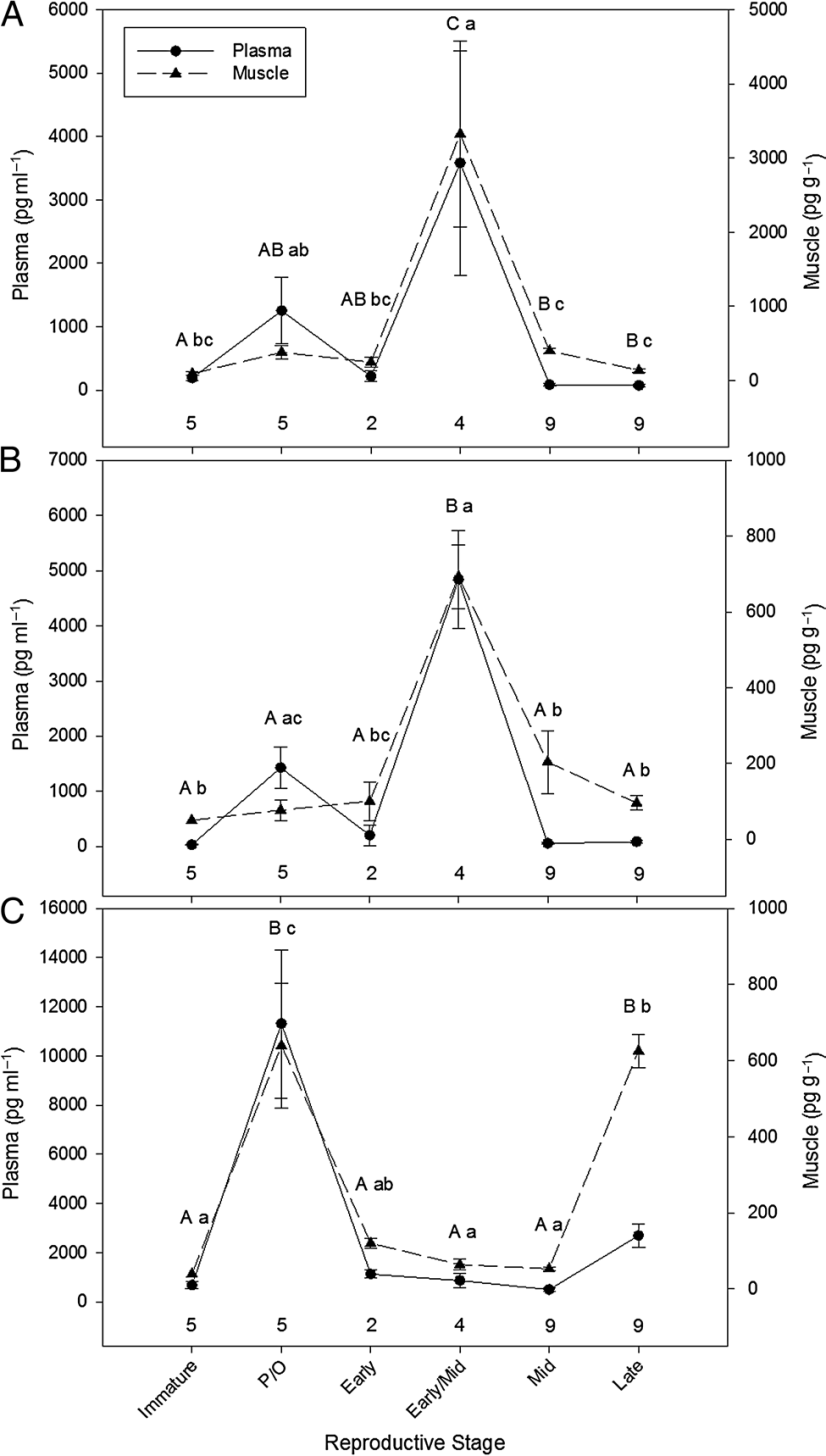
Mean (±SEM) concentrations of plasma (in picograms per millilitre) and muscle steroid hormones (in picograms per gram). Progesterone (**A**), testosterone (**B**), and estradiol (**C**) in *Rhizoprionodon terraenovae*, plotted by reproductive stage [immature, pre-ovulatory (P/O), early gestation, early to mid-gestation, mid-gestation, and late gestation]. Numbers above the *x*-axis represent sample size. Uppercase letters denote statistically significant pairwise differences in muscle hormone concentrations between gestational stages, while lowercase letters denote statistically significant pairwise differences in plasma hormone concentrations between gestational stages (*P* < 0.05). Note the difference in scale between the plasma and muscle axes.

Compared with mature females (Fig. 1a–c), immature female *R. terraenovae* had relatively low concentrations of P_4_, T, and E_2_ in plasma (192 ± 46 pg ml^−1^, *n* = 5; 31 ± 5 pg ml^−1^, *n* = 5; and 680 ± 140 pg ml^−1^, *n* = 5, respectively) and in muscle (95 ± 21 pg g^−1^, *n* = 5; 50 ± 0 pg g^−1^, *n* = 5; and 40 ± 0 pg g^−1^, *n* = 5, respectively).

In mature female *R. terraenovae*, from pre-ovulation to early gestation, plasma and muscle concentrations of P_4_ and T were relatively low. During early to mid-gestation, a significant increase in these hormones was observed, with plasma P_4_ and T concentrations increasing ∼300%, and muscle P_4_ and T concentrations increasing ∼800% (Fig. 1a and b). During mid-gestation, P_4_ and T concentrations decreased significantly by ∼98% in plasma and 75% in muscle (Fig. 1a and b; ANOVA; plasma T, *F*_5,28_ = 20, *P* < 0.0001; and muscle T, *F*_6,27_ = 6.3, *P* < 0.0001). During late gestation, plasma and muscle T and P_4_ concentrations remained unchanged (Fig. 1a and b; ANOVA; plasma P_4_, *F*_5,28_ = 14, *P* < 0.0001; and muscle P_4_, *F*_5,28_ = 15, *P* < 0.0001). Muscle P_4_ and T were significantly correlated with plasma P_4_ and T concentrations (P_4_, linear regression, *r*^2^ = 0.42, *P* < 0.0003; and T, Kendall's τ = 0.43, *P* = 0.0012).

During pre-ovulation, E_2_ concentrations were elevated in plasma and muscle. From early to mid-gestation, plasma and muscle E_2_ concentrations decreased significantly by ∼90%, while in late gestation, E_2_ concentrations increased significantly by 300% in plasma and 850% in muscle (Fig. 1c; ANOVA; plasma E_2_, *F*_5,28_ = 26, *P* < 0.0001; and muscle E_2_, *F*_5,28_ = 63, *P* < 0.0001). *Rhizoprionodon terraenovae* muscle E_2_ concentrations were also found to be significantly correlated with plasma E_2_ (linear regression, *r*^2^ = 0.68, *P* < 0.0001).

Multiple regression analyses revealed that muscle E_2_ concentrations were significant predictors of MFD, OGM, and OM, while a combination of muscle P_4_ and E_2_ concentrations were significant predictors of embryo STL for *R. terraenovae* (Tables 2 and 3).

**Table 2.** Multiple regression analyses conducted on *R. terraenovae*, *Squalus acanthias*, and *Leucoraja erinacea*, investigating the ability of muscle steroid hormones in skeletal muscle tissue to predict reproductive morphology

	Hormones	*r*^2^	*P*-value
*Rhizoprionodon terraenovae*			
MFD (mm)	E_2_	0.90	<0.001
Embryo STL (mm)	E_2_, P_4_	0.84	<0.001
OGM (g)	E_2_	0.77	<0.001
OM (g)	E_2_	0.59	<0.001
*Leucoraja erinacea*			
MFD (mm)	T	0.29	0.0493
OGM (g)	E_2_, P_4_	0.77	0.0115
OM (g)	P_4_, T	0.75	0.0087
*Squalus acanthias*			
MFD (mm)	E_2_, P_4_, T	0.90	<0.001
Embryo STL (mm)	E_2_, P_4_, T	0.75	<0.001
OGM (g)	E_2_, P_4_, T	0.79	<0.001
OM (g)	E_2_, P_4_, T	0.87	<0.001

The relationships between the steroid hormones progesterone (P_4_), testosterone (T), and estradiol (E_2_) and the response variables are indicated by the *r*^2^ and *P*-values. For a more detailed account of the relationships between the hormones and the response variables, see the model definitions in Tables 3, 5, and 6.

**Table 3.** Multiple regression equations generated for *R. terraenovae* using skeletal muscle concentrations (in picograms per gram) of P_4_, T, and E_2_, as indicators of the morphological characteristics

		Gestational stage				
Morphological characteristic	Morphological predictive regression model	Pre-ovulatory	Early	Early to mid	Mid	Late
MFD	MFD = 10.9025 + 8.0295 (E_2_) + 0.7531 [(E_2_)^2^] − 1.1643 [(E_2_)^3^]^a^	20 ± 1.3	4.8 ± 0.9	6.9 ± 1.1	4.3 ± 0.2	19 ± 1.0
Embryo STL	Embryo STL = 138.979 + 2.4.30 (E_2_) − 164.588 (P_4_) − 86.555 [(P_4_)^2^] + 35.556 [(P_4_)^3^] − 142.777 [(E_2_) × (P_4_)]^a^	0	37.7 ± 9.8	66.8 ± 4.2	126 ± 6.8	338 ± 5.7
OM	OM = −23.398 + 6.995 (E_2_)	24 ± 4.5	NA	5.8 ± 1.5	3.6 ± 0.3	20 ± 3.2
OGM	OGM = −1.64748 + 0.70060 (E_2_)	2.7 ± 0.4	NA	1.4 ± 0.3	1.4 ± 0.3	2.9 ± 0.2

The characteristics are included in this table to be utilized as an index, and expressed as mean (±SEM) values for MFD (in millimetres), embryo STL (in millimetres), OM (in grams), and OGM (in grams) by gestational stage. All hormone terms within the regression equations were log transformed. ‘NA’ indicates that data were not collected.

^a^All terms included in the model were centred.

#### Leucoraja erinacea

Fourteen female *L. erinacea* (TL, 38–59 cm; DW, 25–35 cm; 0.34–1.1 kg) were collected and divided into the following reproductive stages: five immature, two mature non-reproductively active (MFD <12 mm), two pre-ovulatory (MFD >19 mm), one ovulatory, with a partly formed egg case, three in pre-oviposition with fully formed egg cases, and one in post-oviposition, having laid an egg case within the previous 12 h (see Table 4 for gestational stage details; Koob *et al.*, 1986; Koob and Callard, 1999). The overall mean recoveries of plasma and muscle P_4_, T, and E_2_ were 51, 83, and 60% and 61, 50, and 68%, respectively. The mean intra-assay coefficients of variation for *L. erinacea* plasma and muscle P_4_, T, and E_2_ assays were 10, 7, 4% and 5, 11, and 9%, respectively. The mean inter-assay coefficients of variation for plasma P_4_ and E_2_ assays (no inter-assay coefficient of variation was calculated for T because all samples were run in one assay) were 12 and 12% for *L. erinacea* plasma assays, respectively, and 13, 12, and 13% for P_4_, T, and E_2_ muscle assays, respectively.

**Table 4.** *Leucoraja erinacea* morphological data

Stage	OM (g)	MFD (mm)	OGM (g)	*n*
Immature	1.5 ± 0.4	2.4 ± 1.0	0.5 ± 0.3	5
Mature	5.7 ± 1.2	9.5 ± 0.7	4.1 ± 0.3	2
Pre-ovulatory	5.9 ± 0.9	21 ± 1.6	5.5 ± 1.9	2
Ovulating	12	22	12	1
Pre-oviposition	8.6 ± 2.3	18 ± 0.9	6.7 ± 0.2	3
Post-oviposition	4.3	12	6.2	1

Values are means (±SEM), by reproductive stage; *n* represents sample size.

**Figure 2.**
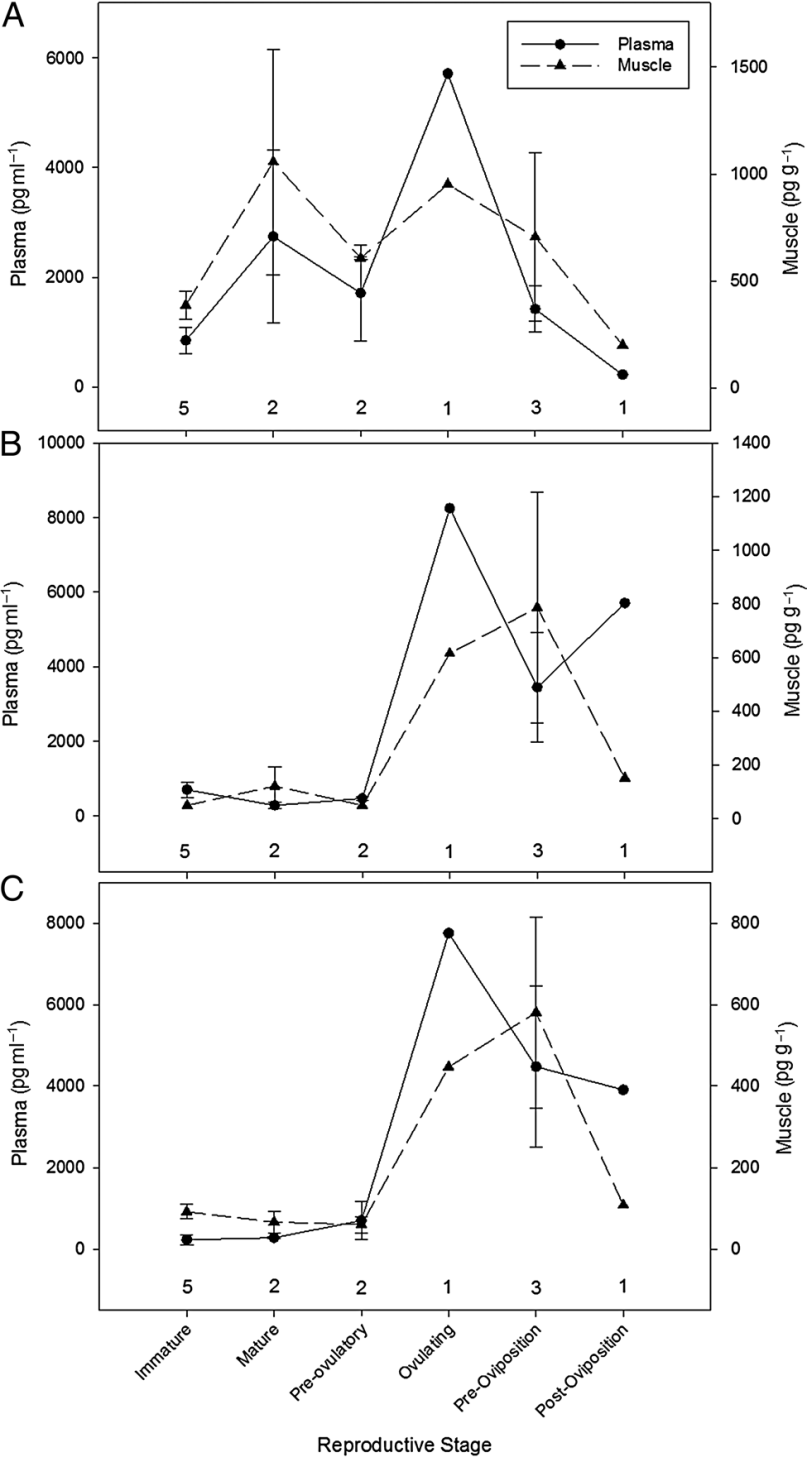
Mean (±SEM) concentrations of plasma (in picograms per millilitre) and muscle steroid hormones (in picograms per gram). Progesterone (**A**), testosterone (**B**), and estradiol (**C**) in *Leucoraja erinacea*, plotted by reproductive stage [immature, mature (non-reproductively active), pre-ovulatory, ovulating, pre-oviposition, and post-oviposition]. Numbers above the *x*-axis represent sample size. Note the difference in scale between the plasma and muscle axes.

Compared with mature female *L. erinacea* (Fig. 2a–c), immature females had relatively low concentrations of P_4_, T, and E_2_ in plasma (846 ± 246 pg ml^−1^, *n* = 5; 710 ± 204 pg ml^−1^, *n* = 5; and 226 ± 123 pg ml^−1^, *n* = 5, respectively) and in muscle (387 ± 64 pg g^−1^, *n* = 5; 50 ± 0 pg g^−1^, *n* = 5; and 91 ± 18 pg g^−1^, *n* = 5, respectively).

Plasma and muscle P_4_ concentrations were elevated in mature, non-reproductively active females and decreased ∼50% at the pre-ovulatory stage (Fig. 2a). Progesterone concentrations increased by 400 and 60% in plasma and muscle, respectively, during ovulation, which was followed by a 75% decrease in plasma and 25% decrease in muscle during pre-oviposition. Plasma and muscle P_4_ concentrations continued to decrease by ∼80% during post-oviposition (Fig. 2a). Similar to P_4_ in *R. terraenovae*, *L. erinacea* muscle P_4_ concentrations were significantly correlated with plasma P_4_ (linear regression, *r*^2^ = 0.35, *P* = 0.022).

In mature, non-reproductively active and pre-ovulatory females, T and E_2_ concentrations were relatively low in plasma and muscle. An elevation in T and E_2_ was noted during ovulation, increasing by ∼1500% in plasma and 500% in muscle (Fig. 2b and c). During pre-oviposition, plasma T and E_2_ concentrations decreased by ∼50%, while muscle T and E_2_ increased by ∼30%. Muscle T and E_2_ concentrations continued to decrease by ∼80% during post-oviposition, while plasma T increased by 66%, and plasma E_2_ decreased by 13% (Fig. 2b and c). Similar to P_4_, *L. erinacea* muscle T and E_2_ concentrations were significantly correlated with plasma T and E_2_ (linear regression, T, *r*^2^ = 0.60, *P* = 0.0011; and E_2_, *r*^2^ = 0.76, *P* < 0.0001).

Muscle T concentrations were significant indicators of MFD, while the combination of muscle P_4_ and E_2_ concentrations were significant indicators of OGM. In addition, the combination of muscle P_4_ and T concentrations were significant indicators of OM for *L. erinacea* (Tables 2 and 5).

**Table 5.** Multiple regression equations generated for *L. erinacea* using skeletal muscle concentrations (in picograms per gram) of P_4_, T, and estradiol, as indicators of morphological characteristics

		Reproductive stage					
Morphological characteristic	Morphological predictive regression model	Immature	Mature	Pre-ovulatory	Ovulating	Pre-oviposition	Post-oviposition
MFD	MFD = −5.125 + 3.531 (T)	2.4 ± 1.0	9.5 ± 0.7	201 ± 1.6	22	18 ± 0.9	12
OM	OM = 5.2419 + 2.1120 (E_2_) + 7.8459 (P_4_) + 0.6632 [(P_4_)^2^] − 5.9695 [(P_4_)^3^]^a^	1.5 ± 0.4	5.7 ± 1.2	5.9 ± 0.9	12	8.6 ± 2.3	4.3
OGM	OGM = 3.7762 + 8.8563 (P_4_) + 1.5244 (T) + 2.3690 [(P_4_)^2^] − 9.0439 [(P_4_)^3^]^a^	0.5 ± 0.3	4.1 ± 0.3	5.5 ± 1.9	12	6.7 ± 0.2	6.2

The characteristics are included in this table to be utilized as an index, and expressed as mean (±SEM) values of MFD (in millimetres), OM (in grams), and OGM (in grams) by reproductive stage. All hormone terms within the regression equations were log transformed.

^a^All terms included in the model were centred.

#### Squalus acanthias

A total of 31 female *S. acanthias* (FL, 78–90 cm; 2.7–4.9 kg) were sampled by Prohaska *et al.* (2013), and additional analyses, including one-way ANOVA and multiple regression analyses, were conducted on these individuals in the present study. The individuals sampled were previously assigned to the following gestational stages: six pre-ovulatory, six containing candles (fertilized follicles enveloped in a thin membrane within the uterus), five early gestation (containing embryos of STL 62–88 mm), eight mid-gestation (containing embryos of STL 190–240 mm), and six late gestation (containing embryos of STL 250–275 mm). The overall mean recoveries of plasma and muscle P_4_, T, and E_2_ were 70, 87, and 80% and 20, 23, and 21%, respectively. The mean intra-assay coefficients of variation for *S. acanthias* plasma and muscle P_4_, T, and E_2_ assays were 6, 7, and 7% and 8, 10, and 8%, respectively. The mean inter-assay coefficients of variation for *S. acanthias* plasma and muscle P_4_, T, and E_2_ assays were 12, 11, and 10% and 12, 11, and 10%, respectively (Prohaska *et al.*, 2013).

**Figure 3.**
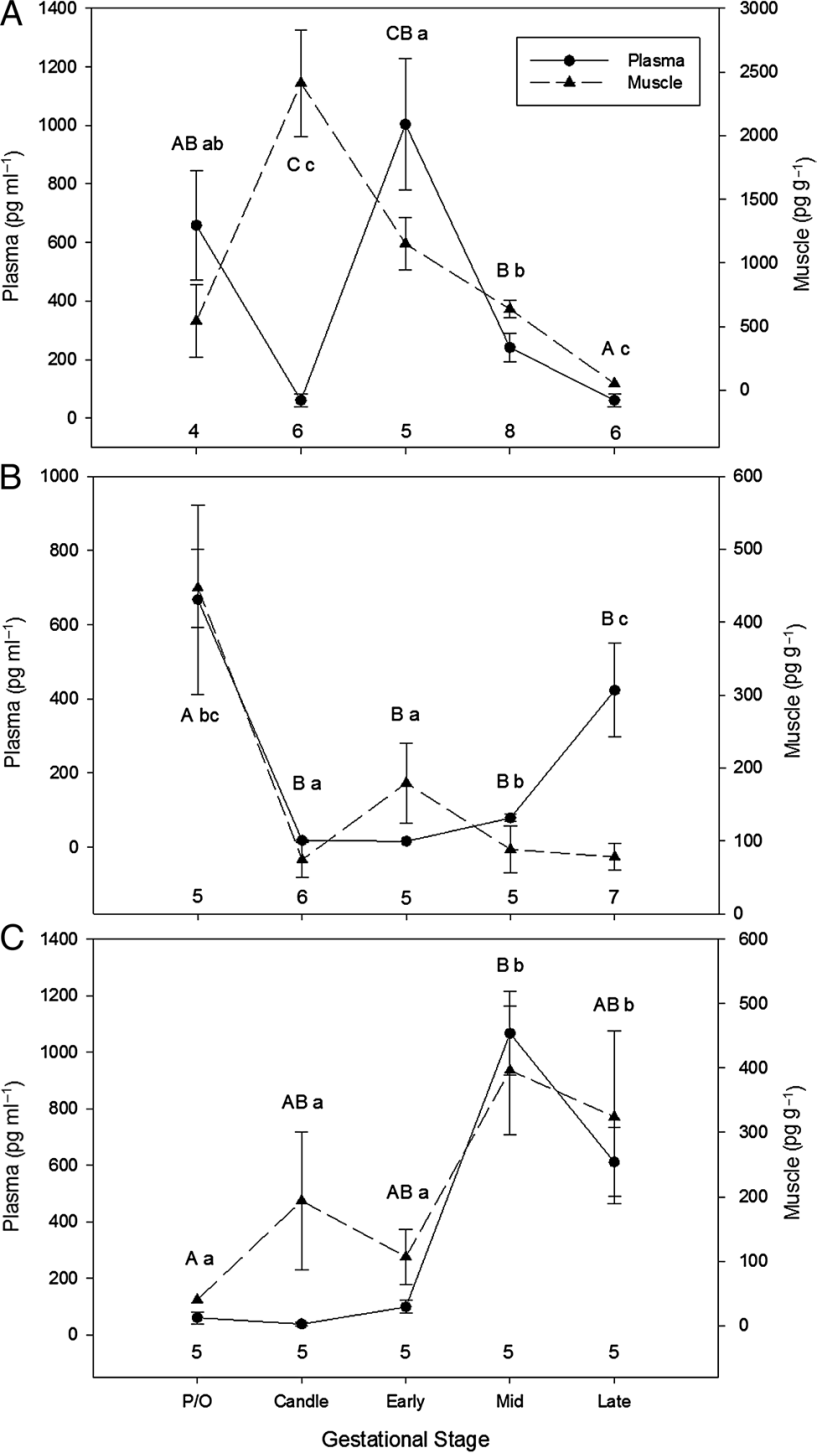
Mean (±SEM) concentrations of plasma (in picograms per millilitre) and muscle steroid hormones (in picograms per gram). Progesterone (**A**), testosterone (**B**), and estradiol (**C**) in *Squalus acanthias*, plotted by reproductive stage [pre-ovulatory (P/O), candle, early gestation, mid-gestation, and late gestation]. Numbers above the *x*-axis represent sample size. Uppercase letters denote statistically significant pairwise differences in muscle hormone concentrations between gestational stages, while lowercase letters denote statistically significant pairwise differences in plasma hormone concentrations between gestational stages (*P* < 0.05). Note the difference in scale between the plasma and muscle axes. This figure is reprinted from Prohaska *et al.* (2013) Development of a non-lethal and minimally invasive protocol to study elasmobranch reproduction. Mar Coast Fish 5: 181–188, by permission of Taylor and Francis (http://www.tandfonline.com); however, the significant pairwise differences in hormone concentrations between gestational stages, denoted by letters, have been added, because this was a new analysis in the present study.

As mature female *S. acanthias* progressed from pre-ovulation to the candle stage of gestation, there was a significant 350% increase in muscle P_4_ and a significant 90% decrease in plasma P_4_ concentrations (Fig. 3a). Plasma P_4_ concentrations increased significantly by 1500% during the early stage of gestation, while muscle P_4_ remained relatively unchanged. From mid-gestation to late gestation, plasma and muscle P_4_ concentrations decreased significantly by ∼70% (ANOVA [P_4_] in plasma, *F*_4,24_ = 17.12, *P* < 0.0001; and muscle, *F*_4,24_ = 22.42, *P* < 0.0001; Fig. 3a). During pre-ovulation, muscle T concentrations were elevated, and then they decreased significantly by ∼90% in both plasma and muscle at the candle stage (Fig. 3b). Muscle T concentrations remained relatively low for the remainder of gestation, while plasma T increased significantly by 100 and 800% to mid-gestation and late gestation, respectively (ANOVA [T] in plasma, *F*_4,23_ = 20.86, *P* < 0.0001; and muscle, *F*_4,23_ = 11.11, *P* < 0.0001; Fig. 3b). Pre-ovulatory plasma and muscle E_2_ concentrations were relatively low, and remained unchanged until plasma concentrations increased significantly by 1200% and muscle concentrations increased by 200% during mid-gestation (ANOVA [E_2_] in plasma, *F*_4,19_ = 33.28, *P* < 0.0001; and muscle, *F*_4,19_ = 3.964, *P* = 0.0167; Fig. 3c).

Similar to *R. terraenovae* and *L. erinacea*, multiple regression analyses showed that a combination of muscle P_4_, T, and E_2_ concentrations were significant indicators of MFD, OM, OGM, and embryo STL in *S. acanthias* (Tables 2 and 6).

**Table 6.** Multiple regression equations generated for *S. acanthias* using skeletal muscle concentrations (in picograms per gram) of P_4_, T, and E_2_, as indicators of morphological characteristics

		Gestational stage				
Morphological characteristic	Morphological predictive regression model	Pre-ovulatory	Candle	Early	Mid	Late
MFD	MFD = −2.649 − 2.073 (E_2_) + 73.115 (P_4_) − 2.883 (T) − 43.726 [(P_4_)^2^)] − 15.840 [(P_4_)^3^] + 10.372 [(P_4_)^4^] + 6.921 [(T)^2^] + 3.018 [(T) × (P_4_)] − 5.547 [(E_2_) × (T)]	44.8 ± 1.5	10.0 ± 0.4	15.6 ± 1.2	33.8 ± 0.6	44.9 ± 1.2
Embryo STL	Embryo STL = 191.060 + 52.658 (E_2_) − 58.531 (P_4_) − 38.695 (T) − 28.323 [(P_4_)^2^] + 30.251 [(E_2_) × (T)]	0	<10.0	76.8 ± 5.0	222 ± 8.1	261 ± 5.5
OM	OM = 148.648 + 1.365 (E_2_) + 74.879 (P_4_) − 125.265 (T) − 58.755 [(E_2_)^2^] + 14.208[(P_4_)^2^] − 25.966 [(P_4_)^3^] − 130.792 [(T)^2^] + 105.000 [(T)^3^] − 52.844 [(E_2_) × (T)]	111 ± 9.6	28.4 ± 12.9	17.0 ± 2.3	62.9 ± 7.7	129 ± 21.9
OGM	OGM = 1.79569 − 0.26788 (E_2_) − 0.32555 (P_4_) + 0.15810 (T) − 0.43729 [(E_2_) × (T)]	2.9 ± 0.3	1.2 ± 0.1	1.1 ± 0.1	1.3 ± 0.1	2.4 ± 0.2

These characteristics are included in this table to be utilized as an index, and expressed as mean (±SEM) values of hormone concentrations, MFD (in millimetres), embryo STL in millimetres), OM (in grams), and OGM (in grams) by gestational stage. All hormone terms within the regression equations were log transformed and centred.

## Discussion

In non-elasmobranch vertebrates, sex steroid hormones are successfully extracted from bodily materials, such as muscle, faeces, and urine, to study reproductive biology (e.g. Lasley and Kirkpatrick, 1991; Heppell and Sullivan, 2000; Shimizu, 2005; Barnett *et al.*, 2009). In addition, fluctuations of hormones present in these bodily materials mirror those in plasma (e.g. Hoffmann and Rattenberger, 1977; Heppell and Sullivan, 2000; Shimizu, 2005; Barnett *et al.*, 2009). For example, studies have quantified P_4_ from blubber of marine mammals to assess whether a female is mature and/or pregnant (Mansour *et al.*, 2002; Kellar *et al.*, 2006). Additionally, urinary and faecal E_2_, T, and P_4_ have been used to track gestation in free-ranging terrestrial vertebrates for conservation purposes (Lasley and Kirkpatrick, 1991; Shimizu, 2005). In teleost fishes, fluctuations in skeletal muscle T and E_2_ concentrations are associated with maturity, sex, and reproductive cycles (Heppell and Sullivan, 2000; Barnett *et al.*, 2009). Previous research on a few species of elasmobranchs reported that circulating E_2_, T, and P_4_ concentrations are correlated with morphological changes in the reproductive tract and with specific events that occur during sexual maturation and the reproductive cycle (e.g. Koob *et al.*, 1986; Rasmussen and Murru, 1992; Sulikowski *et al.*, 2007). More recently, Prohaska *et al.* (2013) successfully extracted E_2_, T, and P_4_ from the skeletal muscle tissue of elasmobranchs and showed that the profiles of these hormones in the muscle are similar to those in the plasma.

Steroid hormones are essential for reproduction. Plasma E_2_ concentrations are primarily linked to the growth and maturation of ovarian follicles in elasmobranchs (e.g. Sumpter and Dodd, 1979; Manire *et al.*, 1995; Snelson *et al.*, 1997, Heupel *et al.*, 1999; Tricas *et al.*, 2000). Medium-sized follicles produce the highest concentrations of E_2_ (Callard and Koob, 1993), which is transported to the liver, where binding to receptors stimulates vitellogenesis (e.g. Sumpter and Dodd, 1979; Manire *et al.*, 1995; Snelson *et al.*, 1997; Heupel *et al.*, 1999; Tricas *et al.*, 2000) and subsequent accumulation of yolk within developing follicles (Perez and Callard 1989; Koob and Callard, 1999). In addition, E_2_ also plays a prominent role in the reproductive tract (Callard *et al.*, 1989), such as enlargement of the oviducal gland (Koob *et al.*, 1986) and vascularization of the uterus (Koob and Callard, 1999). Likewise, in the present study, we found that ovarian follicular growth accompanied increases in plasma and muscle E_2_ concentrations. For example, *R. terraenovae* plasma and muscle E_2_ increased significantly from mid-gestation to late gestation, which corresponded to a substantial increase in follicle diameter. A similar association was observed in the yolk-sac placental viviparous species, *Sphyrna tiburo*, in which serum E_2_ was elevated during pre-ovulation, reduced throughout the majority of gestation, and increased again during the final stages of gestation when follicular growth was beginning (Manire *et al.*, 1995). In *S. acanthias*, a yolk-dependent viviparous species, plasma and muscle E_2_ concentrations increased from early to mid-gestation, and remained elevated for the duration of gestation, paralleling follicular growth (Prohaska *et al.*, 2013). These results are similar to those of Tsang and Callard (1987), who previously reported that plasma E_2_ increased during the second half of gestation when cohorts of ovarian follicles with larger diameters began to develop. Thus, we anticipated that the highest concentrations of plasma and muscle E_2_ would occur in *L. erinacea* at pre-ovulation, during the period of peak follicular development (Koob *et al.*, 1986), but this was not observed in the present study*.* This E_2_ peak might not have been detected because of the high individual variability of hormone concentrations in continuously reproducing oviparous species (Williams *et al.*, 2013). Alternatively, the skates sampled could have been at the end of their follicular growth phase and/or at the beginning of their ovulatory phase. Furthermore, low sample size may account for our inability to detect this peak.

Unlike E_2_, changes in P_4_ are unique to reproductive mode and often linked to mode-specific reproductive events in elasmobranchs. For example, in *S. tiburo*, peaks in serum P_4_ concentrations during early gestation are related to uterine compartmentalization and implantation (Schlernitzauer and Gilbert, 1966; Callard *et al.*, 1992; Manire *et al.*, 1995). In the present study, the peak in plasma and muscle P_4_ during early to mid-gestation for *R. terraenovae* is also likely to be related to uterine compartmentalization and implantation, especially as implantation occurs when embryos are ∼70–85 mm in length (Castro and Wourms, 1993). In *S. acanthias*, high concentrations of plasma P_4_ during early gestation are linked to embryo retention (Callard *et al.*, 1992) and suppression of vitellogenesis (Paolucci and Callard, 1998; Koob and Callard, 1999). Similar to those studies, Prohaska *et al.* (2013) showed that *S. acanthias* plasma and muscle P_4_ peaked during early gestation when little to no follicular growth was occurring, and then decreased into late gestation when follicular growth was beginning. Despite the high individual variability in *L. erinacea* plasma P_4_ concentrations (Williams *et al.*, 2013), the patterns exhibited by plasma and muscle P_4_ in the present study were similar to those found by Koob *et al.* (1986), who noted that plasma P_4_ peaks during ovulation, and then decreases during pre- and post-oviposition. In addition, Koob *et al.* (1986) suggested that the rise in P_4_ might be related to ovulation and the formation of egg cases, while the decrease in P_4_ could be related to oviposition. Furthermore, decreases in P_4_ after ovulation, like those found in the plasma and muscle in the present study, are suggested to inhibit the early release of egg cases by hormonally controlling and tightening cervix muscles to maintain them within the uterus to undergo sclerotization and tanning (Koob and Cox, 1988; Koob and Callard, 1999).

There is a close relationship between T and E_2_ in yolk-dependent viviparous and oviparous elasmobranchs. Plasma T is primarily linked to the growth of follicles (Koob *et al.*, 1986; Tsang and Callard, 1987), with larger follicles producing the highest concentrations, which serve as a substrate for E_2_ synthesis to facilitate vitellogenesis (Tsang and Callard, 1992) and the continued accumulation of yolk by developing follicles (Perez and Callard, 1989; Koob and Callard, 1999). In the present study, the patterns of plasma and muscle T concentrations in *L. erinacea* were similar to those reported in the aforementioned studies, i.e. increases in T are concurrent with increases in follicle diameter (Koob *et al.*, 1986; Tsang and Callard, 1987). However, in the yolk-sac placental viviparous *R. terraenovae*, plasma and muscle T and P_4_ had similar profiles, with both hormones peaking during early to mid-gestation. These profiles are exactly like the ones exhibited in *S. tiburo* serum, in which fluctuations of T were analogous to P_4_, peaking in early gestation when implantation and compartmentalization are suggested to occur (Manire *et al.*, 1995).

Of the few studies so far that determined plasma sex steroid hormones in immature elasmobranchs, all have reported little to no detectable concentrations of these hormones in fishes classified as immature, based on length as well as the underdeveloped condition of reproductive organs (e.g. Rasmussen and Gruber, 1990; Rasmussen and Murru, 1992; Cicia *et al.*, 2009). In the present study, we attempted to measure plasma and muscle P_4_, E_2_, and T concentrations in viviparous and oviparous elasmobranchs that were identified as immature, based on the presence of underdeveloped reproductive tracts. Similar to previous reports, plasma P_4_, E_2_, and T were low compared with those individuals that were mature. In the present study, we also found that the same is true for muscle hormone concentrations. These results provided further support for the direct link between sex steroid hormones, the readiness of the reproductive tract, and reproductive status in elasmobranchs (Rasmussen and Murru, 1992; Gelsleichter *et al.*, 2002; Sulikowski *et al.*, 2006, 2007), as well as the potential use of the muscle hormones to assess maturity.

In addition to examining the relationships between muscle and plasma steroid hormones, multiple regression analyses were conducted in the present study to determine whether skeletal muscle P_4_, T, and E_2_ concentrations relate to OGM, OM, and MFD in *R. terraenovae*, *L. erinacea*, and *S. acanthias*, as well as to embryo STL in the two species of sharks. So far, such analyses have led to the creation of several mathematical models, although the sample sizes used to generate them were low. Nonetheless, these preliminary models are a first step towards developing the use of P_4_, T, and E_2_ concentrations in non-lethally obtained skeletal muscle as indicators of reproductive status and gestational stage.

### Conclusions

The present study reported the successful detection of steroid hormones in the skeletal muscle of female *R. terraenovae*, *L. erinacea*, and *S. acanthias* during specific stages of their reproductive cycles. More importantly, the profiles of plasma and skeletal muscle concentrations of P_4_, T, and E_2_ in *R. terraenovae* and *L. erinacea* were significantly associated, indicating that skeletal muscle tissue is an appropriate substitute for plasma. The present results also affirm the efficacy of using steroid hormones in non-lethally obtained skeletal muscle to assess reproductive status in elasmobranchs (Prohaska *et al.*, 2013). The primary advantage of utilizing muscle vs. blood is that obtaining a small muscle tissue sample may be less stressful, because it does not require that the animal be restrained or removed from the water, and can be obtained more rapidly than blood. While there are caveats in using this method on smaller species, in the long run, skeletal muscle can be used as an alternative when blood cannot be obtained, which will facilitate its use on large species, as well as threatened species of elasmobranchs. Our previous work on *S. acanthias* and *R. terraenovae* suggests that P_4_, T, and E_2_ can be extracted from white skeletal muscle tissue (Prohaska *et al.*, 2013), and the present study further suggests that white skeletal muscle tissue of the oviparous *L. erinacea* can also be used to extract the same three hormones. The analysis of *L. erinacea* skeletal muscle steroid hormones, so far, suggests that they may relate well to reproductive status. However, increasing sample size will strengthen and provide a more accurate depiction of these skeletal muscle hormones throughout the reproductive cycle of an oviparous species. Additionally, the findings of the present study provide a more complete understanding of fluctuations in P_4_, T, and E_2_ during the gestation of *R. terraenovae*, reinforcing our claim that skeletal muscle steroid hormones relate well to gestation in a yolk-sac placental species.

Finally, multiple regression analyses conducted on *R. terraenovae*, *L. erinacea*, and *S. acanthias* suggest that skeletal muscle P_4_, T, and E_2_ are significantly related to changes in reproductive tract morphologies during specific stages of the reproductive cycles including gestation. The robustness of these mathematical models will be strengthened by increasing sample size before they become tools for fisheries managers.
